# Retroperitoneal Lipoblastoma With Cord Compression in an Adult Patient: A Case Report

**DOI:** 10.7759/cureus.29292

**Published:** 2022-09-18

**Authors:** Hosam A Alghanmi, Ammar Bokhari, Ahmed Zeeneldin, Mohammed Y Almaghrabi, Ehab Sadek, Firdos Saba

**Affiliations:** 1 Department of Medical Oncology, Oncology Center, King Abdullah Medical City, Makkah, SAU; 2 Department of Radiation Oncology, Oncology Center, King Abdullah Medical City, Makkah, SAU; 3 Department of Specialized Surgery, Surgical Oncology Section, King Abdullah Medical City, Makkah, SAU; 4 Department of Laboratory, Histopathology Section, King Abdullah Medical City, Makkah, SAU

**Keywords:** retroperitoneal, cord compression, adult, retroperitoneal lipoblastoma, case report

## Abstract

Lipoblastoma is an extremely rare disease and mostly affects the infant to pediatric age group. Primarily, it involves the extremities, head and neck region, and rarely, the retroperitoneal region. Retroperitoneal lipoblastoma mainly presents with either abdominal pain or distension. We report a case of a 24-year-old male who presented with acute cord compression and underwent decompression and fixation with biopsy. Histopathology established the presence of lipoblastoma. Further staging workup showed no metastasis. Immunostaining and cytogenetics were positive for CD34 and negative for desmin, DNA damage-inducible transcript 3 (DDIT3), mouse double minute 2 (MDM2)(12q15), and pleomorphic adenoma gene 1 (PLAG1). The patient underwent resection of the retroperitoneal mass with intraoperative radiation therapy to the tumor bed. The histopathology identified the mass as retroperitoneal lipoblastoma. The postoperative course was uneventful and CT images showed no recurrence or metastasis. This was a unique case of retroperitoneal lipoblastoma in an adult with a unique clinical presentation.

## Introduction

Lipoblastoma is considered a rare benign soft tissue tumor of fat origin [1]. Although benign like lipoblastoma, lipoblastomatosis differs from it with its infiltrative nature [2]. The majority of patients are young infants or children, with most cases occurring during the first three years of life [1,3]. Approximately 70% of all lipoblastomas develop in the head and neck, extremities, trunk, and rarely, the retroperitoneum/mesentery or intrascrotal regions [1,4]. Lipoblastoma in young adults may also be found in the thigh muscles, skin, scrotum, foot, and mediastinum [5-7]. However, retroperitoneal lipoblastoma constitutes less than 5% of all lipoblastomas [1]. Retroperitoneal lipoblastoma frequently presents as a painless mass [3]. Adult patients with lipoblastoma are extremely rare. One reported case was discovered accidentally following abdominal trauma in a 23-year-old male patient. Another report mentioned similar cases in adults who underwent surgical resection as the main treatment [3-7]. A third study was of lipoblastoma in an adult reported as a scrotal mass treated by surgical resection [8]. Our case is unique because of the adult age and the presentation with cord compression.

## Case presentation

**Figure 1:**
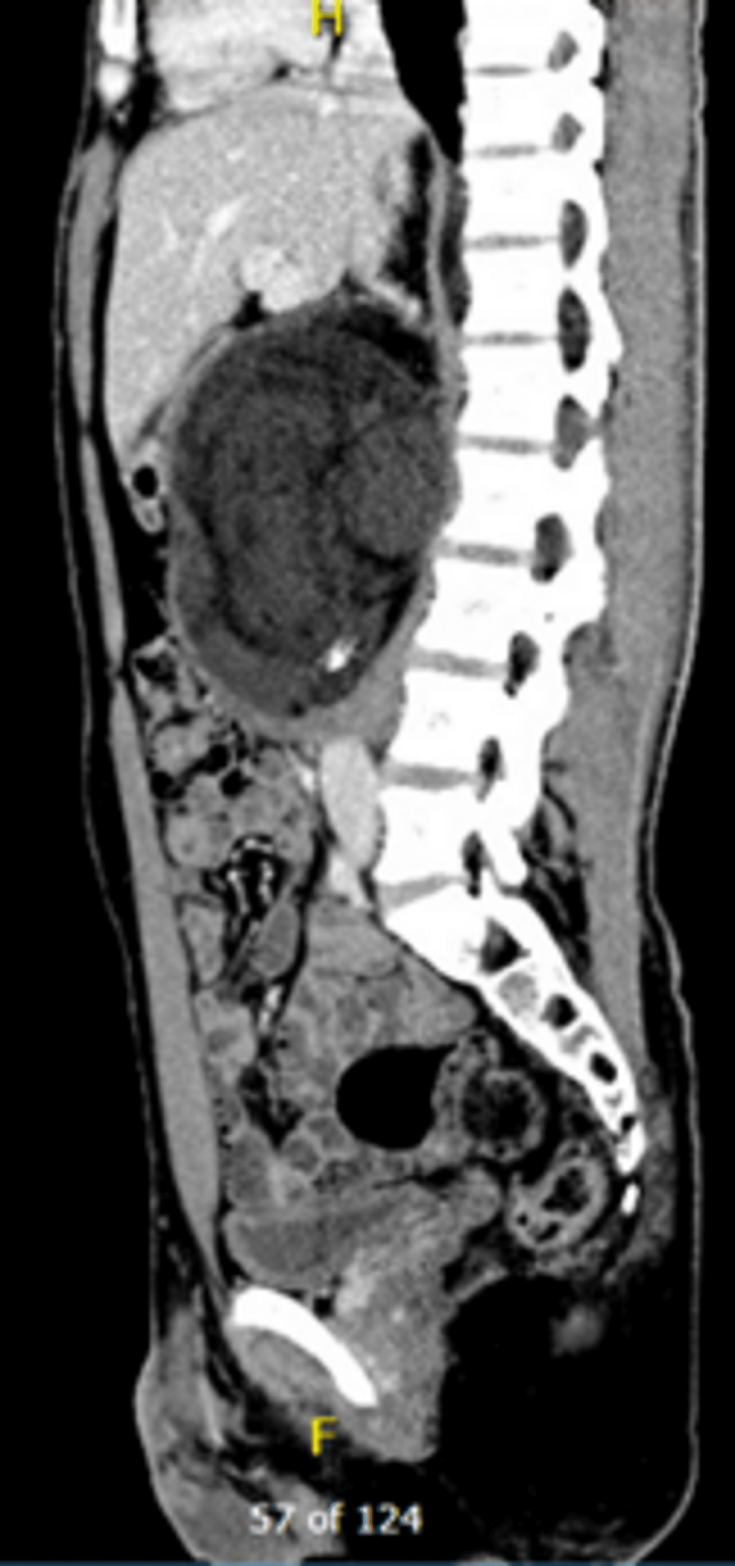
Sagittal view of the retroperitoneal mass

**Figure 2:**
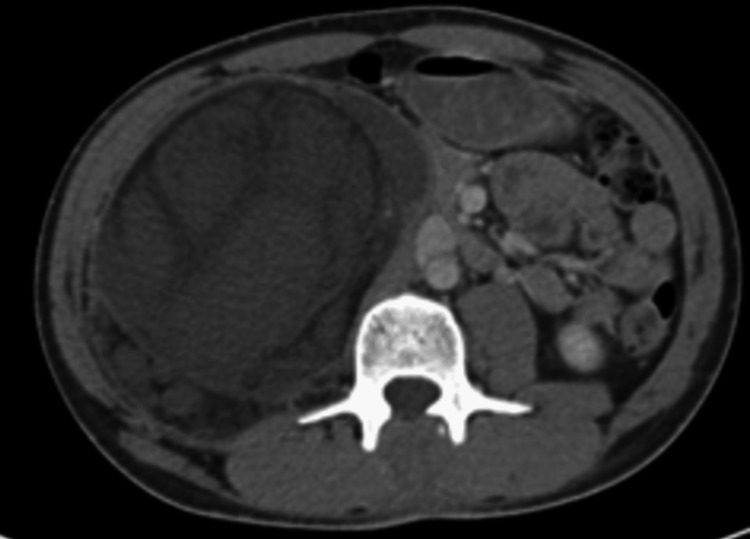
Axial view of the retroperitoneal mass

A 24-year-old male with no medical history presented to the primary hospital complaining of lower back pain lasting for one year and localized without any radiation, partially responsive to analgesics. He had no other neurological symptoms. The patient suddenly developed bilateral lower limb weakness. Further physical examination showed stable vital signs, lower back tenderness, and almost zero power in the lower limbs, with no lower limb sensation and no positive planter upward flexion; other neurological examination results were unremarkable. The patient sought medical advice in a nearby hospital and further workup showed spinal cord compression from the retroperitoneal mass (Figures 1, 2).

**Figure 3:**
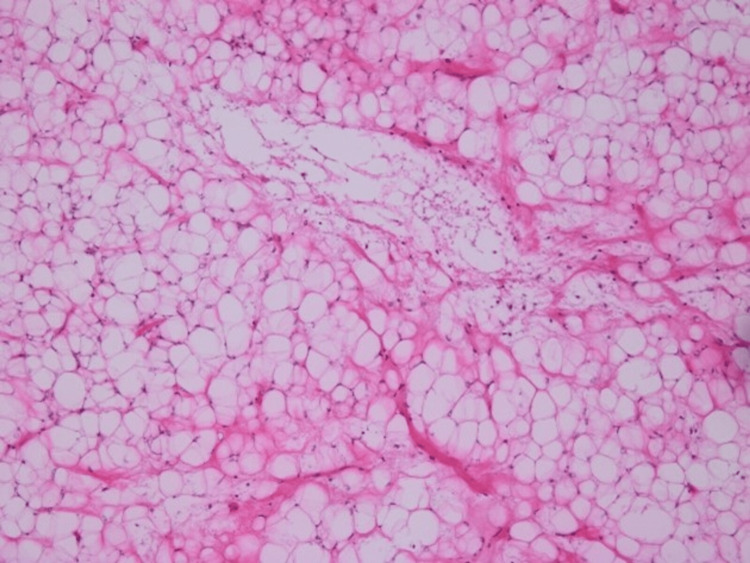
Histopathology showed a myxoid tumor that was moderately cellular and monomorphic without atypia

**Figure 4:**
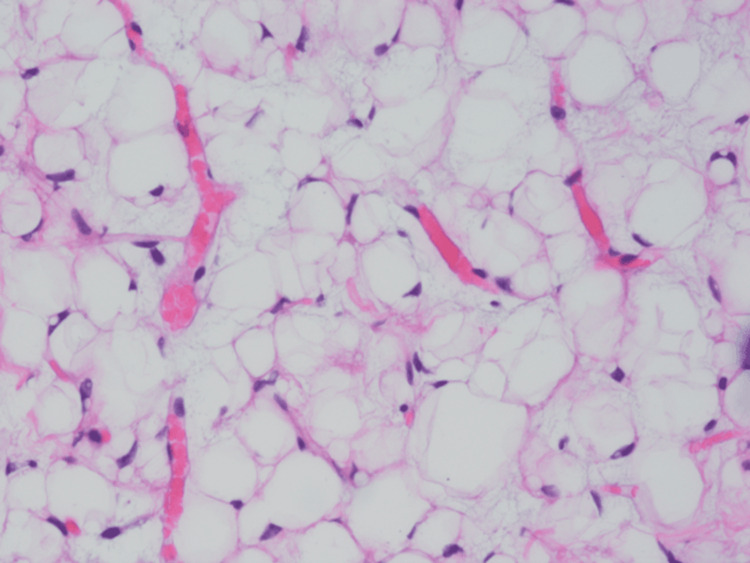
Prominent plexiform vascular like lipoblastoma, positive for CD34 and negative for DDIT3, MDM2, desmin, and PLAG1 fusion genes DDIT3: DNA damage-inducible transcript 3; MDM2: mouse double minute 2; PLAG1: pleomorphic adenoma gene 1.

He was referred to a higher center where he underwent emergency decompression and laminectomy of L1-L3 with gross total excision of the intraspinal component of the tumor. A workup and histopathology were done to rule out other causes of cord compression from infectious and non-infectious causes. Test results for all infectious causes were negative. After decompression surgery, the patient’s weakness and pain were alleviated. While waiting for the histopathology results, an imaging analysis was done to rule out metastasis; the results were negative. Histopathology showed a myxoid tumor that was moderately cellular and monomorphic without atypia (Figures 3, 4).

Further tests were done to rule out malignancies such as liposarcoma and myxoid liposarcoma. Fluorescence in situ hybridization (FISH) results were negative for DNA damage-inducible transcript 3 (DDIT3) and mouse double minute 2 (MDM2). Immunostaining and cytogenetics were positive for CD34 and negative for pleomorphic adenoma gene 1 (PLAG1) fusion and desmin. Based on the previous results, the most important possibility to rule out was liposarcoma. The diagnosis of lipoblastoma requires a PLAG1 fusion-positive specimen, but the result was negative due to the presence of necrosis in the specimen. However, in terms of overall clinical and histopathological results, the diagnosis was compatible with lipoblastoma.

**Figure 5:**
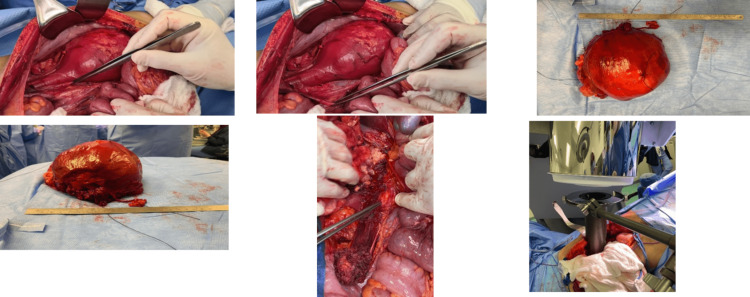
Intraoperative images showing the mass and the area after resection of the mass, with the administration of intraoperative radiation

A tumor board discussion was conducted to set up a treatment plan, and the panel advised the patient to undergo surgical resection and intraoperative radiotherapy, as the tumor was large and it was difficult to achieve a negative margin. The patient later underwent resection of the retroperitoneal lipoblastoma with intraoperative radiotherapy delivery (10 grays) and later external beam radiotherapy was added with 20 grays per five fractions (Figure 5).

**Figure 6:**
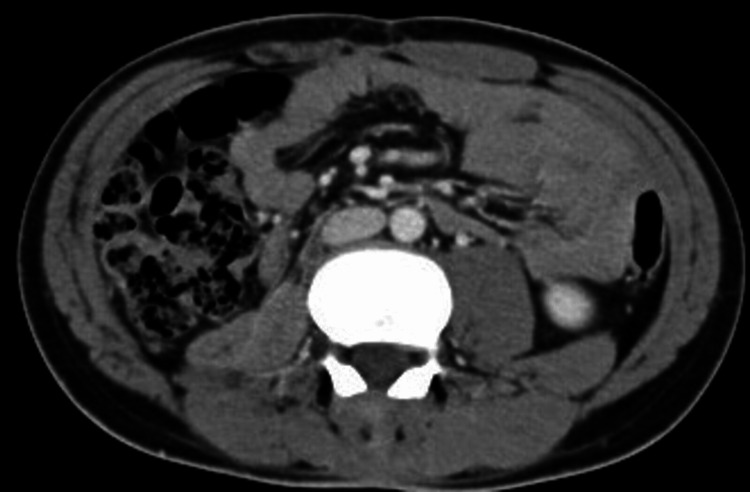
Axial view post-treatment

**Figure 7:**
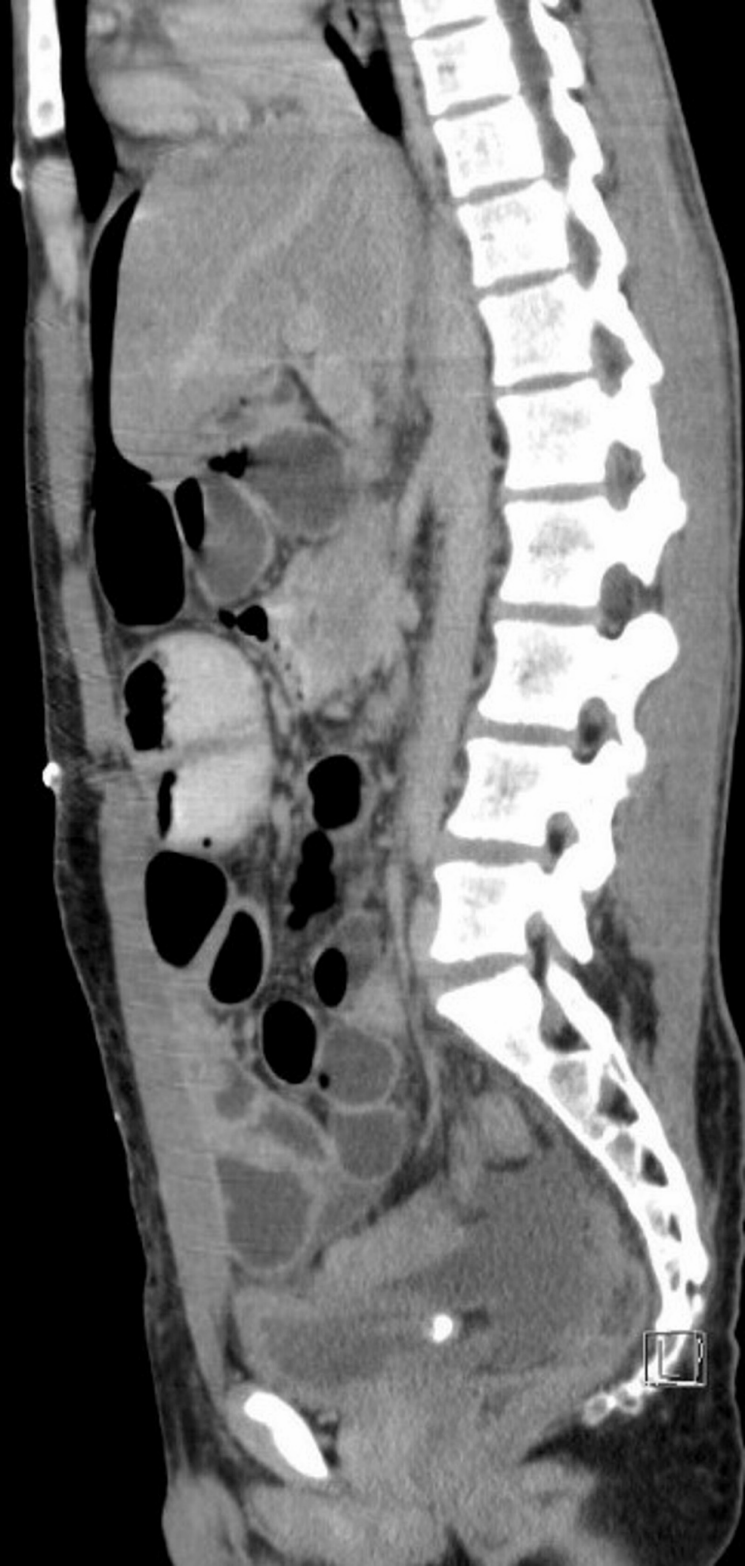
Sagittal view post-treatment

The postoperative course was uneventful, resulting in full recovery. Currently, on follow-up with oncology, radiation oncology, and surgery, the patient is fully functioning without recurrence or neurological deficiencies. A post-treatment follow-up compared to baseline showed the treatment outcome (Figures 6, 7).

## Discussion

Due to a wide spectrum of differential diagnoses for retroperitoneal masses, further histopathology and cytogenetic assessments are required for definitive diagnosis. Due to the similarities between lipoblastoma, lipomatous tumor, and liposarcoma, cytogenetic testing is important to differentiate it from other fat tumors. Lipoblastoma is reportedly associated with changes in the long arm of chromosome 8, with or without PLAG1 re-arrangement [1]. A center-based examination of six cases of adult lipoblastoma with FISH analysis showed classical morphology of lipoblastoma; two cases were positive for PLAG1 and negative for MDM2 amplification [7].

A previous report of similar cases of retroperitoneal lipoblastoma indicated that the main treatment was surgery with maximum resection [1,9]. Management of such rare tumors is guided by previous experience or extrapolated from experience with the treatment of soft tissue sarcoma of the extremities, as there are no clear guidelines. The National Comprehensive Cancer Network recommended surgical resection for retroperitoneal soft tissue sarcoma as it is associated with an excellent impact on local recurrence and survival [10,11]. Hence, the main prognostic factors for mortality in retroperitoneal soft tissue sarcoma are stage at diagnosis, unresectable primary tumor, and grade histology [11].

Due to the acute initial presentation, the patient underwent emergency spinal cord decompression and fixation to save the spinal cord. Later, after evaluation of the case by a multidisciplinary tumor board, the patient was advised to go for surgical resection of the retroperitoneal lipoblastoma. Maximum resection of the retroperitoneal lipoblastoma was very difficult to achieve due to it enclosing the spinal cord, with a high risk for spinal cord injury during surgery. The patient underwent surgery with an uneventful postoperative course and complete recovery of neurological symptoms. The inability to perform a complete resection is considered a poor prognostic factor; therefore, another treatment modality should be included as there is no role for chemotherapy in the case of lipoblastoma to further decrease the risk of recurrence. Adding either neoadjuvant, intraoperative, or adjuvant radiation therapy would help to decrease local recurrence, but with no survival benefit [12].

Intraoperative radiation (IORT) boost compared to external beam radiation (EBRT) boost spared all radiosensitive structures, minimized radiation exposure, and had the benefit of a short treatment course. The drawbacks of this approach are that the pathological margin cannot be assessed for treatment success, and the use of a high single dose might result in increased late toxicity [13].

A retrospective study of 908 patients with retroperitoneal soft tissue sarcoma demonstrated that adding IORT to EBRT showed increased survival benefit compared to IORT or EBRT alone, with a survival benefit of 87 months from the combined modality compared to 34 months with one of the modalities alone [12].

Retroperitoneal lipoblastoma is extremely rare in adults. Its treatment approach was extrapolated from retroperitoneal soft tissue sarcoma where the cornerstone of treatment is surgical resection, achieving a negative margin. Additional combined radiation therapy (IORT + EBRT) is recommended to decrease the local risk of recurrence if a clear margin or negative gross tumor cannot be achieved. Further, large-scale clinical trials are needed to elucidate the benefits and toxicity, as there is conflicting data based on retrospective studies and small trials.

## Conclusions

Retroperitoneal lipoblastoma is extremely rare in adults. The treatment approach was extrapolated from retroperitoneal soft tissue sarcoma. The cornerstone of treatment is surgical resection, achieving a negative margin. Additional combined radiation therapy (IORT + EBRT) is recommended to decrease the local risk of recurrence if a clear margin or negative gross tumor cannot be achieved.
